# Low Copy Number of the *AMY1* Locus Is Associated with Early-Onset Female Obesity in Finland

**DOI:** 10.1371/journal.pone.0131883

**Published:** 2015-07-01

**Authors:** Heli Viljakainen, Johanna C Andersson-Assarsson, Miriam Armenio, Minna Pekkinen, Maria Pettersson, Helena Valta, Marita Lipsanen-Nyman, Outi Mäkitie, Anna Lindstrand

**Affiliations:** 1 Children's Hospital, University of Helsinki and Helsinki University Hospital, Helsinki, Finland; 2 Department of Molecular and Clinical Medicine, Sahlgrenska Academy, University of Gothenburg, Gothenburg, Sweden; 3 Center for Cardiovascular and Metabolic Research, Sahlgrenska Academy, University of Gothenburg, Gothenburg, Sweden; 4 Department of Molecular Medicine and Surgery, and Center for Molecular Medicine, Karolinska Institutet, Stockholm, Sweden; 5 Folkhälsan Institute of Genetics, Helsinki, Finland; 6 Department of Clinical Genetics, Karolinska University Hospital, Stockholm, Sweden; Estonian Biocentre, ESTONIA

## Abstract

**Background:**

The salivary α-amylase locus (*AMY1*) is located in a highly polymorphic multi allelic copy number variable chromosomal region. A recent report identified an association between *AMY1* copy numbers and BMI in common obesity. The present study investigated the relationship between *AMY1* copy number, BMI and serum amylase in childhood-onset obesity.

**Patients:**

Sixty-one subjects with a history of childhood-onset obesity (mean age 19.1 years, 54% males) and 71 matched controls (19.8 yrs, 45% males) were included. All anthropometric measures were greater in the obese; their mean BMI was 40 kg/m^2^ (range 25-62 kg/m^2^) compared with 23 kg/m^2^ in the controls (15-32 kg/m^2^).

**Results:**

Mean *AMY1* copy numbers did not differ between the obese and control subjects, but gender differences were observed; obese men showed the highest and obese women the lowest number of *AMY1* copies (p=0.045). Further, only in affected females, *AMY1* copy number correlated significantly with whole body fat percent (r=-0.512, p=0.013) and BMI (r=-0.416, p=0.025). Finally, a clear linear association between *AMY1* copy number and serum salivary amylase was observed in all subgroups but again differences existed between obese males and females.

**Conclusions:**

In conclusion, our findings suggest that *AMY1* copy number differences play a role in childhood-onset obesity but the effect differs between males and females. Further studies in larger cohorts are needed to confirm these observations.

## Introduction

Human obesity is a complex disorder affected by many interacting genetic and non-genetic factors. In the past decade genetic studies have increased our understanding of the mechanisms controlling energy balance. First, through studies on patients and animal models with rare single gene defects in which obesity is the main phenotypic manifestation, several pathways and hormones regulating hunger and lipid metabolism have been identified [1]. This work has highlighted the importance of genes in the leptin pathway (*LEP*, *LEPR*, *POMC*, *PCSK1*, *MC4R)* [2–7] and recently homozygous stop mutations have also been identified in *CEP19*, encoding the cilia protein CEP19 in a large family with recessive obesity [8]. Second, genome-wide association studies have linked many common genetic variants—each with a weak association—to an increased risk of adiposity and obesity [9]. In addition, epigenetic mechanisms due to DNA methylation or histone modification of DNA in gene regulatory regions may play a role in the pathogenesis of obesity [10,11]. Lifestyle factors, especially high energy intake and low or sedentary physical activity, ethnic origin and socioeconomic status have a major influence [12]. Even though progress has been made in this way, the genetic factors contributing to childhood-onset obesity are still poorly characterized.

Genomic copy number variants (CNVs) have emerged as important contributors to the genetic load in various disorders and several observations have suggested that CNVs may contribute to the heritability of complex diseases and common traits [13]. The salivary α-amylase locus (*AMY1*) is located in a tandem CNV region on human chromosome 1p21.1, with the reported number of gene copies ranging from two to 20 [14,15]. A strong correlation between the number of gene copies and serum amylase levels has been demonstrated [16]. Further, a link with the human dietary evolution from a low to a high starch diet has been suggested with higher *AMY1* counts in societies with higher starch consumption [16]. Two recent reports have identified an association between *AMY1* copy number and common obesity [14,17] with lower number of gene copies associated with an increased body mass index (BMI). This link between carbohydrate metabolism and obesity is novel since previously known “obesity-genes” are mainly expressed in the brain and involved in appetite regulation [18]. However, the association is well supported by previous papers demonstrating that consumption quick carbohydrates such as sugar-sweetened beverages associates positively with measures of obesity and homeostasis model assessment (HOMA) index [19,20].

These recent observations prompted us to study whether *AMY1* copy numbers differ between individuals with severe early-childhood onset obesity and non-obese control subjects. Furthermore we investigated whether the variation in *AMY1* copy number associates with serum amylase fractions and total amylase levels as well as with other biochemical and clinical characteristics in these obese and non-obese subjects.

## Materials and Methods

### Ethics Statement

The Research Ethics Committee of the Hospital District of Helsinki and Uusimaa approved the study. All study participants or guardians of subjects younger than 18 years of age gave an informed written consent.

### Study subjects

This study was designed to assess metabolic, skeletal, and genetic characteristics of severe childhood-onset obesity. Patient recruitment and assessment were carried out at Children's Hospital, Helsinki University Central Hospital, Finland. Inclusion criteria for the subjects with early-onset severe obesity were: i) weight-for-height exceeding 60% before 7 years of age; according to Finnish growth standards this value defines severe, early-onset obesity, ii) referral due to severe obesity to Children's Hospital, Helsinki University Central Hospital, during childhood, iii) living in the greater Helsinki area at age 7 years, and iv) aged between 15 and 25 years at the time of recruitment. All obese participants had been followed at Children's Hospital and endocrine and common disorders underlying obesity had been excluded (e.g. Prader Willi syndrome, pseudohypoparathyroidism, hypercortisolism, hypothyroidism). Age- and sex-matched controls were selected from the national population register; similar to obese subjects, the controls were from the greater Helsinki area. Altogether 61 subjects (93% ethnic Finns; two subjects were half Finnish and two subjects, of non-Finnish origin) and 71 normal weight controls (100% ethnic Finns) were included. None of the controls had been obese before or during puberty. All obese and control individuals met the inclusion criteria, however at the time of study visit a few obese subjects had lost weight and a few controls had gained weight. Obese and control subjects were included in the present study if blood samples for DNA and serum analyses were available. Excluded subjects (N = 9) were similar in age (p = 0.74), BMI (p = 0.3), waist circumference (p = 0.2), and gender distribution (p = 0.55) to the participants.

### Clinical assessment

Data on lifestyle factors was collected with a questionnaire. Anthropometry including height, weight, and waist and hip circumferences was collected during the study visit, as described previously [21].

### Laboratory measurements

Blood samples were obtained after an over-night (at least 10 hours) fast. Plasma total amylase and pancreatic amylase were measured according to International Federation of Clinical Chemistry recommendations using a kinetic photometric method on Roche Modular P analyzer (Roche Diagnostics, Germany). The analysis of pancreatic amylase was based on inhibition of salivary amylase isoenzyme activity by two monoclonal antibodies. The concentration of salivary amylase was calculated for the present analysis by subtracting the pancreatic amylase from the total amylase concentration. Fasting plasma glucose was analyzed by spectrophotometric hexokinase and glucose-6-phosphate dehydrogenase assay (Gluko-quant glucose/hexokinase, Roche Diagnostics) with an automated Hitachi Modular. Impaired fasting glucose was defined as plasma glucose concentration >6.1 but <7.0 mmol/l. Diabetes was defined as fasting plasma glucose concentration ≥7.0 mmol/l at 0 min [22]. All subjects were considered normoglycemic. All serum samples were centrifuged and divided into aliquots and stored at -80°C for further analyses.

Serum insulin was measured with a time-resolved immunofluorometric assay (Perkin Elmer Life Sciences, Finland) with a detection limit of 0.5 mU/l and an interassay CV less than 4%. The insulin-resistance index determined by HOMA was calculated as the product of the fasting serum insulin concentration (in mU/l) and fasting plasma glucose concentration (in mmol/l) divided by 22.5 [23]. The glycosylated hemoglobin (HbA1c) was measured by photometric immunoassay.

### Assessment of body composition

Whole body (WB) Dual-energy X-ray absorptiometry (DXA) with the Lunar Prodigy Advance instrument (GE Healthcare Lunar, Madison, WI) was used to determine body composition. Lean mass (LM), total fat mass (FM), trunk FM and android FM were extracted using automated analysis. Data were available for 57 obese and 71 normal-weight controls. DXA for subjects weighing >160 kg became available only in 2013; 6 subjects with severe obesity therefore did not undergo DXA in 2011–2012 and in 2013 two subjects weighing >160 kg were measured with Lunar iDXA from the same manufacturer. Seven subjects did not consent to DXA measurement.

### Droplet digital PCR of the *AMY1* locus

Copy number of the *AMY1* gene was determined using the QX200 droplet digital PCR (ddPCR) system (Bio-Rad Laboratories, Hercules, CA) according to the manufacturer's instructions. Briefly, each reaction consisted of 11 μl ddPCR Supermix for probes (no UTP), 1 μl of *AMY1*-assay (900nM primers and 250nM probe), 1 μl of *AP3B1*-assay (900nM primers and 250nM probe), 1 μl HaeIII (3U/μl), 6 μl dH_2_O, and 2 μl of DNA (10ng/μl). 20 μl of each reaction was transferred to a droplet generation cartridge, 70 μl of Droplet Generation Oil was added and droplets generated in a Droplet Generator. PCR amplification was performed in a Veriti Thermal Cycler (Life Technologies, Waltham, MA) with the following conditions: 10 minutes at 95°C, 40 cycles of 30 seconds at 94°C and 60 seconds at 60°C with ramp speed set to 60%, and 10 minutes at 98°C. After amplification, reactions were stored at 4°C until droplets were read in a QX200 Droplet Reader. A negative control (water) and two samples with known *AMY1* copy number were included in each run. Study samples were analyzed in triplicate. Initial data quality control and analysis was performed using QuantaSoft v1.6.6.0320 (Bio-Rad Laboratories).

### Statistical analyses

Normality of variables was visually inspected in the whole cohort and subgroups. In case of non-normal distribution, logarithmic transformations were used. Comparison of baseline characteristics between groups was performed with either Independent Samples t-test or Mann-Whitney U-test. For categorical variables Chi-Square test was applied. Associations were tested either with Pearson or Spearman correlation, and when adding in possible confounders (age, gender), with partial correlations. Linear regression was used to investigate associations in more detail or to demonstrate interaction. β-coefficient was used to quantify the association and adjusted R^2^ to describe the explanatory rate are given for models. A significant gender by *AMY1* copy number interaction effect was detected in predicting BMI (p = 0.048). Therefore, subgroups based on gender and patient status were created. Mean *AMY1* copy numbers between subgroups were tested with ANOVA including LSD post-hoc test for pairwise comparison, while distributions were tested with Kruskal-Wallis test and Median test.

A p-value of < 0.05 was considered statistically significant, while p-values < 0.1 were considered suggestive. When considering interaction, a p-value < 0.2 was considered meaningful.

## Results

### Background characteristics

The final study cohort consisted of 61 (32 males and 29 females) obese subjects and 71 (32 males and 39 females) controls. The obese and controls were initially age- and sex-matched but due to exclusion of subjects with incomplete data from the present study minor differences between the groups arose (Table 1). Of the obese subjects, 54% were males while the corresponding number in the control group was 45%. The age of subjects did not differ between subgroups. As expected, all anthropometric measures were greater in obese subjects than in the controls. The mean BMI was 40 kg/m^2^ in obese subjects (range 25–62 kg/m^2^) and 23 kg/m^2^ in controls (range 15–32 kg/m^2^). In obese and control males the mean whole body fat% was 41% and 20%, and waist circumference 115 cm and 80 cm, respectively. In females the corresponding numbers in obese subjects and controls were 52% and 32% and 117 cm and 71 cm, respectively.

**Table 1:** Baseline characteristics of subgroups with mean (SD).

	Obese males		Obese females		Control males		Control females		P value
N	32		29		32		39		
Age at enrolment, y	19.1	(2.6)	19.1	(2.5)	19.6	(2.8)	20.0	(2.5)	0.407
Weight, kg	121.1	(30.4)	123.9	(27.2)	75.7	(14.2)	60.8	(7.1)	<0.001^d^
Height, cm	180.1	(6.8)	167.6	(6.4)	181.0	(8.0)	166.7	(6.1)	<0.001
BMI, kg/m^2^	37.3	(9.2)	43.8	(8.0)	23.2	(4.5)	21.9	(2.4)	<0.001^d^
Waist circumference, cm^a^	115.0	(19.5)	117.1	(18.0)	80.2	(10.0)	71.0	(6.9)	<0.001^d^
WB fat% ^b^	41.2	(8.4)	52.0	(5.3)	20.3	(9.6)	32.0	(5.1)	<0.001^d^
fP-Glucose, mmol/l	5.6	(1.5)	5.4	(0.6)	5.3	(0.5)	5.0	(0.4)	0.028
fS-Insulin, mU/l	16.9	(11.6)	18.5	(11.1)	6.7	(3.4)	6.3	(3.5)	<0.001^d^
HOMA index ^c^	4.3	(3.1)	4.5	(2.7)	1.6	(0.9)	1.4	(0.8)	<0.001^d^
Plasma total amylase, U/l	39.6	(11.3)	40.0	(14.9)	50.5	(15.3)	55.7	(17.9)	<0.001
Plasma pancreatic amylase, U/l	18.1	(5.8)	19.2	(6.4)	22.8	(8.0)	24.9	(8.4)	<0.001
Plasma salivary amylase, U/l	21.5	(9.2)	20.8	(11.7)	27.8	(10.8)	30.8	(14.7)	0.001
*AMY1* copy number	7.4	(2.8)	6.1	(2.1)	6.4	(2.1)	6.6	(2.4)	0.205

^a^ N = 30 for obese males

^b^ N = 28 for obese males, N = 23 for obese females

^c^ N = 30, N = 28, N = 31 and N = 38 for obese males, obese females, control males and control females, respectively

^d^ Kruskal-Wallis test

SD = standard deviation, BMI = body mass index, WB = whole body, BP = blood pressure, fP = fasting plasma, fS = fasting serum, HOMA = homeostasis model assessment.

### Biochemical findings

Obese subjects had higher fasting glucose and insulin concentrations, resulting in higher HOMA index, indicating insulin resistance. Abnormally high fasting insulin concentration (>12 mU/l) [24] was observed in 63% of the obese subjects and 4% of the controls. Total, pancreatic and salivary amylase concentrations were 20–30% lower in obese subjects compared with the controls (p<0.001) (Table 1). Within each group concentrations did not differ between males and females (p-values between 0.3 and 0.9). Significant inverse associations were observed between measures of adiposity (BMI, waist circumference and whole body fat%) and concentrations of amylase in the whole cohort, and separately in the obese, but not in control subjects (Table 2). Inverse associations between HOMA and total, pancreatic and salivary amylase concentrations were observed in the entire cohort (p<0.005). This was not seen in the controls alone (p = 0.6), but a suggestive association was observed separately in the obese subjects (p<0.2). Formal statistical significance was likely not reached in the obese subjects due to the small sample size.

**Table 2:** Partial correlation between variables related to amylase and markers of adiposity in all subjects, obese and controls after controlling for gender and age.

		AMY1 CN	ln_BMI	ln_WC	WB fat%	Total Plasma Amylase, U/l	Plasma Panreatic Amylase, U/l	Plasma Saliva Amylase, U/l	ln_HOMA
AMY1 CN	r	-	-0.035	-0.049	-0.008	0.496	0.115	0.578	-0.054
	p	-	0.695	0.583	0.933	< 0.001	0.191	< 0.001	0.553
ln_BMI	r	-0.035	-	0.963	0.896	-0.458	-0.361	-0.382	0.600
	p	0.695	-	< 0.001	< 0.001	< 0.001	< 0.001	< 0.001	< 0.001
ln_WC	r	-0.049	0.963	-	0.868	-0.474	-0.368	-0.396	0.650
	p	0.583	< 0.001	-	< 0.001	< 0.001	< 0.001	< 0.001	< 0.001
WB fat%	r	-0.008	0.896	0.868	-	-0.385	-0.313	-0.311	0.655
	p	0.933	< 0.001	< 0.001	-	< 0.001	< 0.001	0.001	< 0.001
ln_HOMA	r	-0.054	0.600	0.650	0.655	-0.316	-0.235	-0.279	-
	p	0.553	< 0.001	< 0.001	< 0.001	< 0.001	0.008	0.002	-
**OBESE SUBJECTS**									
AMY1 CN	r	-	-0.198	-0.191	-0.188	0.564	0.041	0.681	-0.015
	p	-	0.133	0.154	0.196	< 0.001	0.760	< 0.001	0.914
ln_BMI	r	-0.198	-	0.908	0.857	-0.531	-0.382	-0.442	0.370
	p	0.133	-	< 0.001	< 0.001	< 0.001	0.003	0.001	0.005
ln_WC	r	-0.191	0.908	-	0.742	-0.489	-0.367	-0.394	0.407
	p	0.154	< 0.001	-	< 0.001	< 0.001	0.005	0.002	0.002
WB fat%	r	-0.188	0.857	0.742	-	-0.366	-0.254	-0.308	0.618
	p	0.196	< 0.001	< 0.001	-	0.010	0.079	0.032	< 0.001
ln_HOMA	r	-0.015	0.370	0.407	0.618	-0.197	-0.136	-0.170	-
	p	0.914	0.005	0.002	< 0.001	0.145	0.318	0.212	-
**CONTROLS**									
AMY1 CN	r	-	0.028	-0.030	-0.091	0.547	0.197	0.592	-0.166
	p	-	0.819	0.811	0.463	< 0.001	0.110	< 0.001	0.179
ln_BMI	r	0.028	-	0.839	0.716	-0.019	-0.119	0.045	0.145
	p	0.819	-	< 0.001	< 0.001	0.876	0.337	0.718	0.242
ln_WC	r	-0.030	0.839	-	0.734	-0.144	-0.117	-0.117	0.320
	p	0.811	< 0.001	-	< 0.001	0.245	0.344	0.345	0.008
WB fat%	r	-0.091	0.716	0.734	-	-0.160	-0.204	-0.087	0.305
	p	0.463	< 0.001	< 0.001	-	0.194	0.097	0.483	0.012
ln_HOMA	r	-0.166	0.145	0.320	0.305	-0.074	0.005	-0.099	-
	p	0.179	0.242	0.008	0.012	0.549	0.969	0.425	-

CN = copy number, BMI = body mass index, WC = waist circumference, WB = whole body, HOMA = homeostasis model assessment.

### *AMY1* copy number and association with clinical and biochemical findings

**Fig 1:**
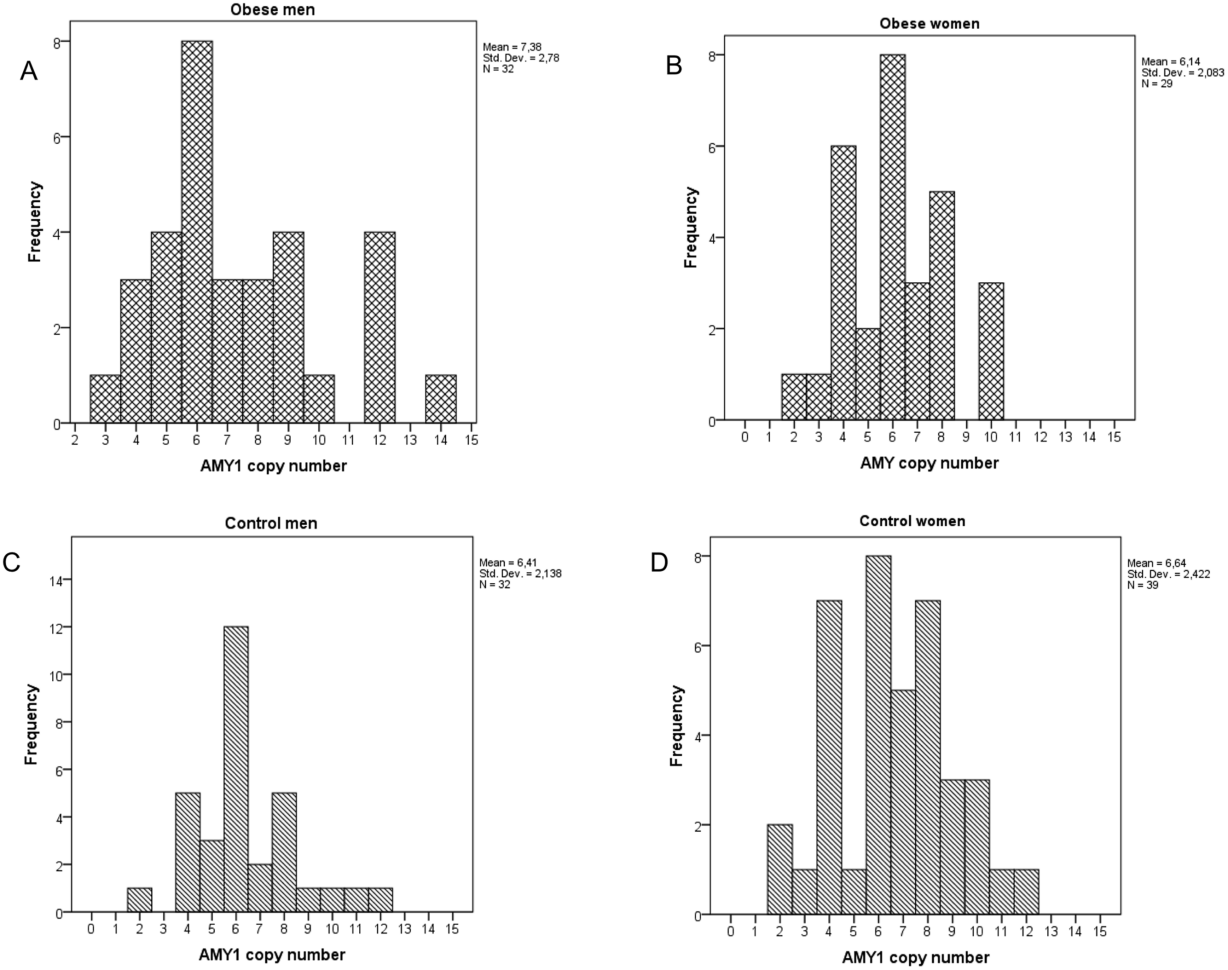
Distribution of copy number of *AMY1* separately in obese men (A) and obese women (B) and in control men (C) and control women (D). Mean values differed between obese men and obese women (ANOVA: LSD p = 0.045).

The distribution of *AMY1* copy number in the four groups of obese and control men and women is shown in Fig 1. The mean *AMY1* copy numbers did not significantly differ between the four subgroups (ANOVA; p = 0.205). However, a post-hoc exploratory analysis revealed that obese men had the highest and obese women the lowest number of copies (cn = 7.38 for men and cn = 6.14 for women; p = 0.045). Although distributions were non-normally divided, the medians and distributions did not differ significantly between subgroups (Independent Samples Media test p = 0.4 and Kruskal-Wallis test p = 0.4).

**Fig 2:**
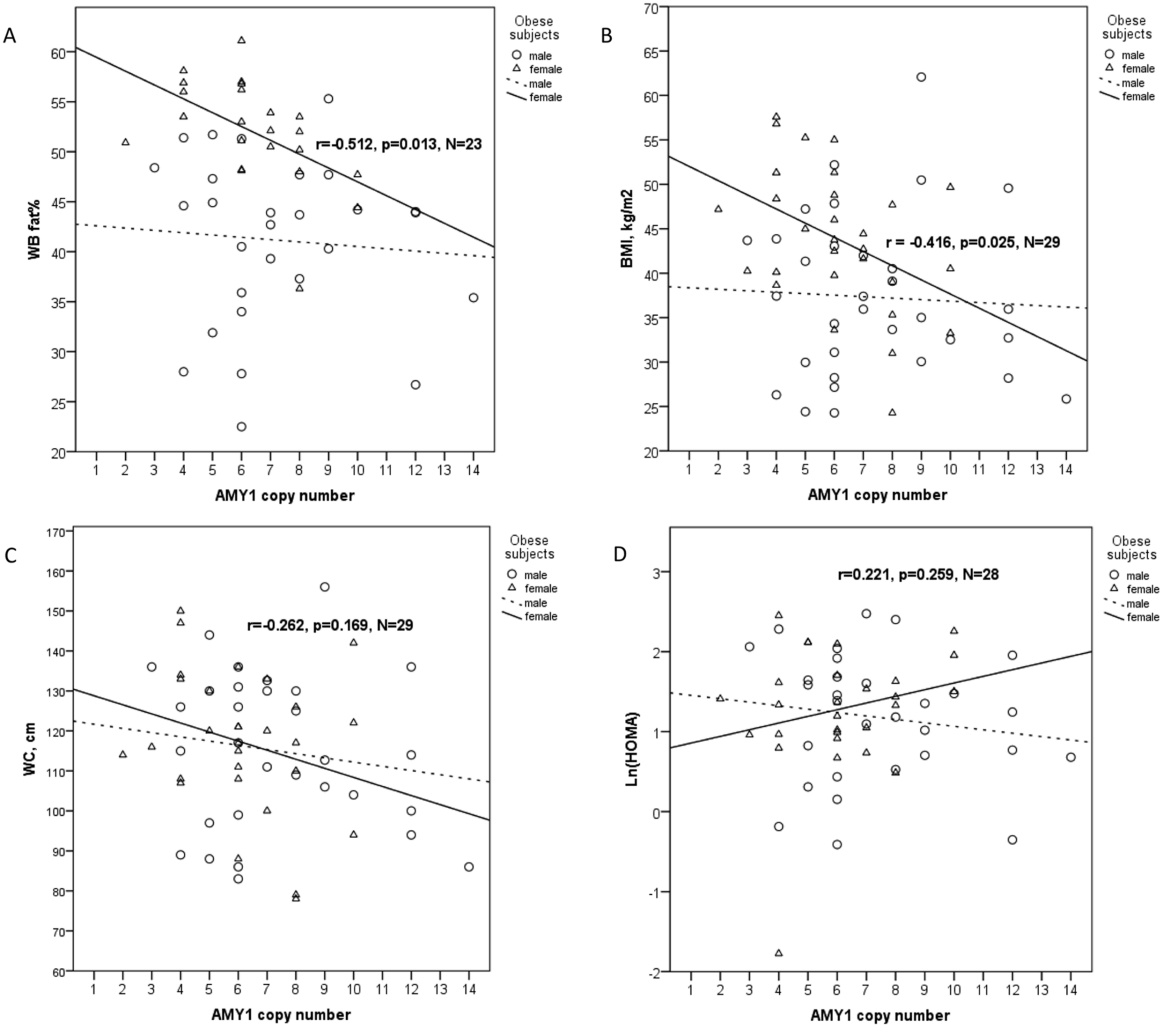
Association between *AMY1* copy number with A) whole body fat%, B) BMI, C) waist circumference and D) HOMA in obese women (triangles) and obese men (circles). Measurements for BMI were obtained at study visit; some subjects with a history of severe childhood obesity had improved BMI.

A significant inverse association between markers of adiposity (fat% and BMI) and *AMY1* copy number was observed in obese females as shown in Fig 2 (r = -0.512, p = 0.013 and r = -0.416, p = 0.025, respectively). We also observed a weak but non-significant positive association between *AMY1* copy number and ln HOMA in obese females (Pearson r = 0.221, p = 0.259). In contrast, no such correlation was observed in normal-weight females or in obese or control males. Partial correlation analysis in all subjects revealed weak, but non-significant, correlations between *AMY1* copy number and BMI, waist circumference, fat% and HOMA (p<0.2 for all, Table 2) in obese subjects.

Some obese subjects had succeeded in weight management (N = 8) while most had persistent obesity (N = 53). The mean (SD) values for *AMY1* copy numbers were 7.8 (3.5) and 6.8 (2.3) (p = 0.365) for weight losers and constantly obese, respectively. The respective values in controls who maintained normal-weight (N = 67) and in controls who became obese (N = 4) were 6.6 (2.3) and 5.5. (1.3) (p = 0.355). Our study does not suggest that higher copy numbers might be related to weight loss in obese persons or to maintenance of normal weight in controls.

**Fig 3:**
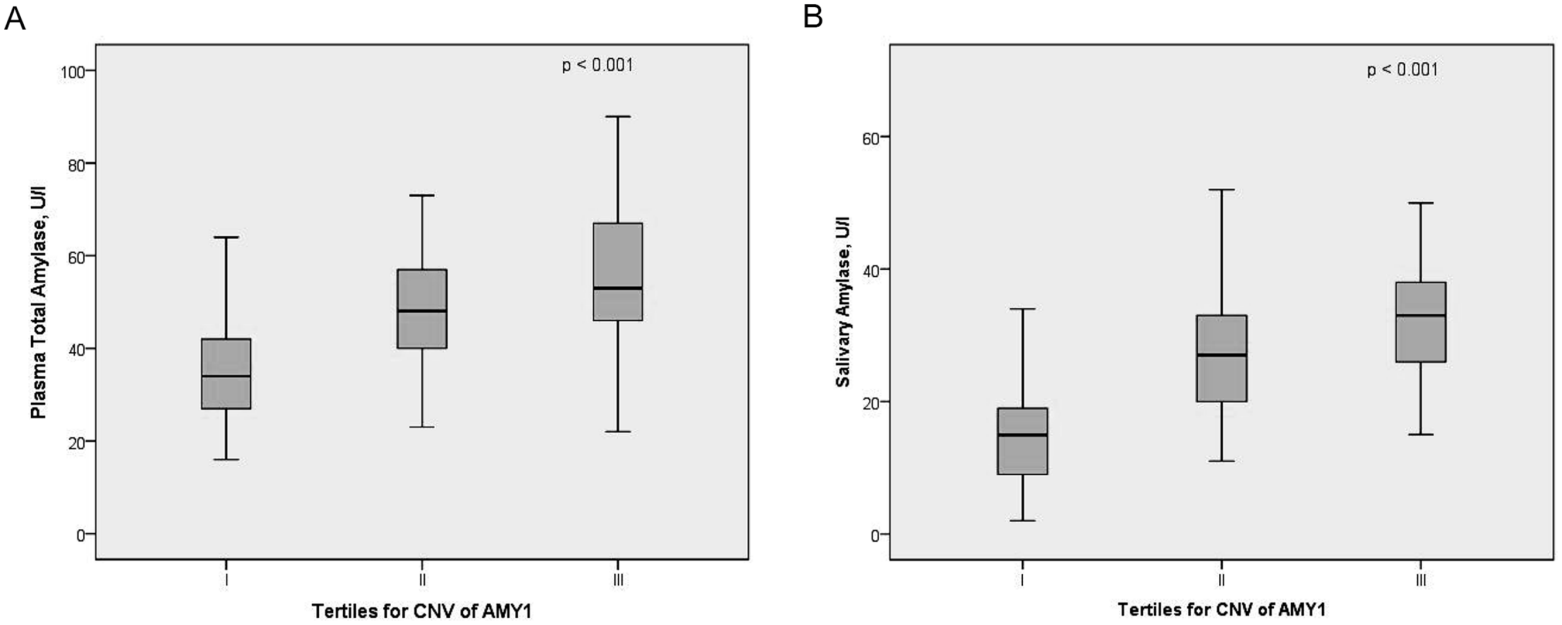
Association of *AMY1* copy number with concentration of total amylase (A) and salivary amylase (B).

Strong positive correlation between *AMY1* copy number and total and salivary amylase concentrations was found both in the whole cohort and in the subgroups separately (Table 2, Fig 3). The association was further explored with linear regression. In the combined dataset each additional gene copy increased the concentration of total amylase by 3.2 U/L [95% CI 2.1–4.2] and salivary amylase by 2.8 U/L [95% CI 2.2–3.7] explaining 21% and 30% of the respective amylase concentrations. However, the *AMY1* copy number status affected total and salivary amylase concentrations differently in the four subgroups. The highest increase rate per copy was noted in obese women (Table 3) (5.1 U/l and 4.3 U/l; explaining 49% and 56%; of total and salivary amylase concentrations, respectively) while the lowest increases were detected in obese men (1.9 U/l and 2.2 U/l per copy; explaining 19% and 41%; of total and salivary amylase concentrations respectively). In controls, the gender interaction with *AMY1* copy number was related more to total than salivary amylase concentration (corresponding p-values for interaction 0.18 and 0.33, respectively). Thus, an increase in *AMY1* copy number increased the total amylase concentration by 3.6 U/l in control men and 4.4 U/l in control women (explaining 22% and 33%, of enzyme levels), while for salivary amylase the corresponding figure was 3.4 U/l for both male and female control subjects (explaining 34% of enzyme levels).

**Table 3:** Association of *AMY1* copy number with total and salivary concentration in the four subgroups (obese men, obese women, control men, control women).

**Model summary on total plasma amylase, U/l**							
Subgroups	Variables	Unstandardized B Coefficients	P value	95% CI for B		Adjusted R^2^	SEE
Obese men	Constant	25.6	< 0.001	15.1	36.1	0.192	10.1
	*AMY1* CN	1.9	0.007	0.6	3.2		
Obese women	Constant	8.9	0.167	-3.9	21.7	0.487	10.6
	*AMY1* CN	5.1	< 0.001	3.1	7.1		
Control men	Constant	27.7	0.001	12.1	43.3	0.223	13.5
	*AMY1* CN	3.6	0.004	1.2	5.9		
Control women	Constant	26.9	< 0.001	12.8	40.9	0.328	14.7
	*AMY1* CN	4.3	< 0.001	2.4	6.3		
**Model summary on salivary amylase, U/l**							
Subgroups	Variables	Unstandardized B Coefficients	P value	95% CI for B		Adjusted R^2^	SEE
Obese men	Constant	5.5	0.138	-1.9	12.9	0.405	7.1
	*AMY1* CN	2.2	< 0.001	1.2	3.1		
Obese women	Constant	-5.5	0.239	-14.8	3.9	0.562	7.8
	*AMY1* CN	4.3	< 0.001	2.8	5.7		
Control men	Constant	8.1	0.112	-2.0	18.2	0.349	8.7
	*AMY1* CN	3.1	< 0.001	1.6	4.6		
Control women	Constant	7.2	0.214	-4.3	18.7	0.328	12.0
	*AMY1* CN	3.6	< 0.001	1.9	5.2		

CI = confidence interval, SEE = standard error of estimate.

## Discussion

Here, we demonstrate a link between the *AMY1* copy number polymorphism and early onset obesity in females. We noted a significant inverse correlation between *AMY1* copy number and whole body fat% and BMI in severely obese young women, while no association was observed in female controls or in male controls or obese cases. We also show that both gender and obesity status influence the biochemical effect of the *AMY1* copy number on total amylase and salivary amylase concentrations with the highest increase per copy noted in obese women and the lowest in obese men. In addition, a weak but non-significant positive association between HOMA index and *AMY1* copy number was identified exclusively in obese women.

The gender difference in *AMY1* copy number and the biochemical effects of variable copy numbers is novel and merits further investigation in additional cohorts. However, it should be noted that there is a small, but significant, difference in BMI between obese women and obese men. Mean BMI was 43.8 in obese women compared to 37.3 in obese men. Earlier studies [14,17] have shown a clear inverse correlation between BMI and *AMY1* copy number and hence, the lower BMI among males could, at least in part, explain the higher mean copy number in this group. There is also more males than females with high number of copies, which is what would be expected if the gender difference is true. It is important to keep in mind though that this is a small cohort and therefore the higher copy number distribution in males may be random.

Previous work has suggested that roughly 30% of serum total amylase (the sum of salivary and pancreatic amylase) is explained by the *AMY1* copy number polymorphism. Further low serum amylase concentrations have previously been observed in both obese humans [14,25] and mice [26] as well as high amylase concentrations in bulimic patients [27]. Here we show that even though there were no differences in mean *AMY1* copy numbers between obese subjects and controls, we observed substantially lower serum amylase concentrations in obese subjects compared with controls (40 U/l vs 53 U/l for cases and controls respectively; p<0.001). Further, our data implies that *AMY1* gene copies are less functional in obese men than in obese women. A single *AMY1* gene copy influences amylase concentrations almost three times more in obese women compared with obese males (β coefficients; 5.1 U/l vs 1.9 U/l for women and men, respectively). This discrepancy creates a paradox; although *AMY1* mean copy number is the highest in obese men (7.4), the effect on amylase concentration is the weakest. The underlying mechanism for this remains unclear but our data emphasizes the importance of genetic variation in determining the amount of α-amylase in saliva.

High *AMY1* copy number and high salivary amylase activity are favorable for the digestion of starchy foods and may even modify individual desires for starchy food as effective digestion with high salivary amylase increases sweetness [28]. We hypothesize that amylase levels may also influence the glycemic response after starch consumption. High salivary amylase activity could be expected to allow faster digestion and thus lead to higher postprandial blood glucose concentration, as seen in studies where oral digestion is fully avoided [29]. However, the opposite has also been reported: that early (preabsorptive) insulin release (within the first 9 minutes) was /quicker/more brisk in subjects with high *AMY1* copy number compared with subjects with low *AMY1* copy number, resulting in lower postprandial glucose levels in healthy, normal-weight women [30]. Our data suggests that low *AMY1* copy number in obese young women might prevent from insulin resistance. Further, a recent study suggested that satiety sensation after consumption of carbohydrate and protein meals is related to the early (postprandial) insulin response specifically in obese women [31].

Because the *AMY1* copy number is associated with serum amylase it is likely to influence glucose metabolism directly by affecting the digestion of carbohydrates, starting in the mouth through salivary amylase. When less salivary amylase is produced then the digestion of starchy carbohydrate rich foods takes longer inducing lower satiety and in consequence the impaired satiety may increase food intake. Starchy foods are prominent components of the human diet, especially among agricultural societies. Earlier studies have shown that people on traditionally low starch diets have lower number of *AMY1* copies than people on western types of diets [16]. Correspondingly, in our cohort the mean copy number was relatively high, on average 6.8 (±2.5), in line with the fact that traditional Finnish diet relies strongly on food items with high starch content such as rye bread, oatmeal porridge and potatoes.

In a broader context the correlation between *AMY1* copy number with serum and salivary amylase levels may elucidate the mechanism linking low copy numbers to female obesity. A high consumption of carbohydrates with high glycemic index has been regarded as a culprit for the increasing weight gain in western populations [32]. Earlier studies have identified an increased risk of obesity in individuals with low *AMY1* copy numbers [14,17]. Our data here suggest that certain women on a western diet are unable to properly digest starchy foods and therefore prone to develop early-onset severe obesity. On the other hand, despite predisposed to adiposity, a possible protective effect against insulin resistance was noted in the same obese women. An explanation for this might be that lazy digestion of starchy foods provides composed elevation of plasma glucose. However, it will be necessary to replicate and confirm these gender-specific correlations in other studies.

In conclusion, although no causal relationships between obesity and *AMY1* copy number can be drawn, we noticed that lower *AMY1* copy number count is a potential risk factor for severe obesity especially in women. Earlier findings on the effects of variable *AMY1* copy number are from large population-based cohorts with thousands of adult obese individuals [14]. Here we investigated the clinical relevance of *AMY1* copy number in a well-characterized, ethnically relatively homogeneous cohort of young adults with severe, early-onset obesity and compared the findings with a group of normal-weight age and sex-matched controls. As the conclusions drawn are limited both by the small cohort size as well as the lack of detailed data on the dietary patterns, the observed gender differences remain to be confirmed in larger cohorts. Further, the association between *AMY1* copy numbers and various metabolic parameters remains to be explored in future studies.

## Supporting Information

S1 Table Clinical, biochemical and genetic data for normal and obese subjects. (DOCX)
