# Supplementary material for: Low Copy Number of the AMY1 Locus Is Associated with Early-Onset Female Obesity in Finland
Source: PLoS One. 2015 Jul 1;10(7):e0131883. doi: 10.1371/journal.pone.0131883 (PMC4489572; doi:10.1371/journal.pone.0131883)
Supplement: S1 Table — (DOCX) [file pone.0131883.s001.docx]

- **S1 Table.** **Clinical, biochemical and genetic data for normal and obese subjects.**

| **Study subject** | **Group;  1= case, 2=control** | **Gender; 1= male, 2=female** | **BMI** | **Whole Body Fat%** | **HOMA index** | **Waist Circumference** | **P_Amyl** | **P_AmylP** | **AMY1 cn** |
| --- | --- | --- | --- | --- | --- | --- | --- | --- | --- |
| 1 | 1 | 2 | 24.27 | 36.3 | 1.62 | 79 | 66 | 36 | 8 |
| 2 | 1 | 1 | 24.28 | 22.5 | 0.66 | 83 | 49 | 22 | 6 |
| 3 | 1 | 1 | 24.42 | 31.9 | 1.36 | 88 | 57 | 35 | 5 |
| 4 | 1 | 1 | 25.85 | 35.4 | 1.97 | 86 | 66 | 20 | 14 |
| 5 | 1 | 1 | 26.31 | 28.0 | 0.83 | 89 | 27 | 17 | 4 |
| 6 | 1 | 1 | 27.17 | 34.0 | 1.17 | No data | 31 | 17 | 6 |
| 7 | 1 | 1 | 28.20 | 26.7 | 0.70 | 94 | 50 | 20 | 12 |
| 8 | 1 | 1 | 28.24 | 27.8 | 1.54 | 86 | 48 | 18 | 6 |
| 9 | 1 | 1 | 29.96 | 44.9 | 4.88 | 97 | 26 | 11 | 5 |
| 10 | 1 | 1 | 30.06 | 40.3 | 2.02 | 106 | 36 | 12 | 9 |
| 11 | 1 | 2 | 30.98 | 48.0 | 4.20 | 78 | 67 | 27 | 8 |
| 12 | 1 | 1 | 31.10 | 40.5 | 4.00 | 99 | 40 | 20 | 6 |
| 13 | 1 | 1 | 32.52 | 44.2 | 4.37 | 104 | 47 | 23 | 10 |
| 14 | 1 | 1 | 32.72 | 44.0 | 2.16 | 100 | 46 | 13 | 12 |
| 15 | 1 | 2 | 33.24 | 44.4 | 4.49 | 94 | 66 | 29 | 10 |
| 16 | 1 | 2 | 33.61 | 48.2 | 3.30 | 88 | 57 | 19 | 6 |
| 17 | 1 | 1 | 33.66 | 37.3 | 3.26 | 109 | 49 | 27 | 8 |
| 18 | 1 | 1 | 34.32 | 35.9 | 4.30 | 117 | 39 | 19 | 6 |
| 19 | 1 | 1 | 35.00 | 47.7 | 2.77 | 113 | 41 | 13 | 9 |
| 20 | 1 | 2 | 35.31 | 53.5 | 5.11 | 110 | 56 | 30 | 8 |
| 21 | 1 | 1 | 35.95 | 39.3 | 11.89 | 111 | 33 | 11 | 7 |
| 22 | 1 | 1 | 35.95 | 43.9 | 3.47 | 114 | 48 | 18 | 12 |
| 23 | 1 | 1 | 37.40 | 42.7 | 2.99 | 133 | 62 | 16 | 7 |
| 24 | 1 | 1 | 37.42 | 44.6 | No data | 115 | 30 | 21 | 4 |
| 25 | 1 | 2 | 38.65 | 56.9 | 2.63 | 107 | 27 | 17 | 4 |
| 26 | 1 | 2 | 39.00 | 50.2 | No data | 117 | 47 | 13 | 8 |
| 27 | 1 | 1 | 39.09 | 47.7 | 1.69 | 125 | 33 | 13 | 8 |
| 28 | 1 | 2 | 39.74 | 51.1 | 8.14 | 108 | 47 | 23 | 6 |
| 29 | 1 | 2 | 40.11 | 53.5 | 5.02 | 108 | 41 | 26 | 4 |
| 30 | 1 | 2 | 40.24 | No data | 2.61 | 116 | 21 | 13 | 3 |
| 31 | 1 | 2 | 40.50 | 47.7 | 7.06 | 122 | 42 | 13 | 10 |
| 32 | 1 | 1 | 40.51 | 43.7 | 11.03 | 130 | 30 | 15 | 8 |
| 33 | 1 | 1 | 41.33 | 47.3 | 2.28 | 130 | 42 | 21 | 5 |
| 34 | 1 | 2 | 41.62 | 52.1 | 2.85 | 120 | 58 | 19 | 7 |
| 35 | 1 | 1 | 41.98 | 43.9 | 4.98 | 130 | 34 | 17 | 7 |
| 36 | 1 | 2 | 42.48 | 57.0 | 2.78 | 117 | 48 | 14 | 6 |
| 37 | 1 | 2 | 42.71 | 53.9 | 2.09 | 100 | 48 | 28 | 7 |
| 38 | 1 | 1 | 43.05 | No data | 6.81 | 126 | 38 | 18 | 6 |
| 39 | 1 | 1 | 43.68 | 48.4 | 7.86 | 136 | 19 | 10 | 3 |
| 40 | 1 | 2 | 43.76 | 53.0 | 5.49 | 121 | 47 | 17 | 6 |
| 41 | 1 | 1 | 43.86 | 51.4 | 9.79 | 126 | 34 | 26 | 4 |
| 42 | 1 | 2 | 44.42 | 50.5 | 4.64 | 133 | 33 | 16 | 7 |
| 43 | 1 | 2 | 44.98 | No data | 8.31 | 120 | 34 | 24 | 5 |
| 44 | 1 | 2 | 46.01 | 48.1 | 2.70 | 136 | 30 | 16 | 6 |
| 45 | 1 | 2 | 47.17 | 50.9 | 4.09 | 114 | 13 | 11 | 2 |
| 46 | 1 | 1 | 47.22 | 51.7 | 5.18 | 144 | 38 | 22 | 6 |
| 47 | 1 | 2 | 47.69 | 52.0 | 3.78 | 126 | 38 | 18 | 8 |
| 48 | 1 | 1 | 47.85 | No data | 5.39 | 136 | 42 | 24 | 6 |
| 49 | 1 | 2 | 48.37 | 56.0 | 2.22 | 134 | 31 | 23 | 4 |
| 50 | 1 | 2 | 48.79 | 56.8 | 3.92 | 111 | 27 | 15 | 6 |
| 51 | 1 | 1 | 49.57 | No data | 7.07 | 136 | 36 | 16 | 12 |
| 52 | 1 | 2 | 49.66 | No data | 9.54 | 142 | 50 | 14 | 10 |
| 53 | 1 | 1 | 50.48 | No data | No data | No data | 22 | 7 | 9 |
| 54 | 1 | 2 | 51.30 | 58.1 | 117.09 | 147 | 26 | 20 | 4 |
| 55 | 1 | 2 | 51.34 | 56.2 | 2.50 | 121 | 27 | 12 | 6 |
| 56 | 1 | 1 | 52.20 | 51.3 | 7.70 | 131 | 23 | 12 | 6 |
| 57 | 1 | 2 | 55.01 | 61.1 | 1.96 | 115 | 33 | 17 | 6 |
| 58 | 1 | 2 | 55.25 | No data | 8.28 | 130 | 16 | 11 | 5 |
| 59 | 1 | 2 | 56.80 | No data | 0.17 | 133 | 36 | 18 | 4 |
| 60 | 1 | 2 | 57.58 | No data | 3.80 | 150 | 28 | 18 | 4 |
| 61 | 1 | 1 | 62.07 | 55.3 | 3.87 | 156 | 50 | 24 | 9 |
| 62 | 2 | 1 | 14.31 | 8.3 | 1.86 | 63 | 48 | 21 | 6 |
| 63 | 2 | 1 | 16.56 | 7.5 | 2.11 | 67 | 79 | 36 | 8 |
| 64 | 2 | 1 | 16.87 | 9.0 | 1.84 | 66 | 18 | 16 | 2 |
| 65 | 2 | 2 | 17.33 | 26.8 | 0.15 | 61 | 76 | 38 | 9 |
| 66 | 2 | 1 | 17.95 | 7.2 | 1.80 | 70 | 48 | 29 | 4 |
| 67 | 2 | 2 | 18.02 | 32.3 | 1.10 | 66 | 20 | 18 | 2 |
| 68 | 2 | 1 | 18.04 | 18.9 | 1.59 | 70 | 50 | 22 | 6 |
| 69 | 2 | 2 | 18.07 | 35.4 | 0.92 | 67 | 35 | 23 | 4 |
| 70 | 2 | 1 | 18.34 | 16.6 | No data | 81 | 57 | 10 | 8 |
| 71 | 2 | 2 | 18.71 | 29.7 | 1.03 | 69 | 43 | 18 | 4 |
| 72 | 2 | 2 | 18.92 | 22.7 | 2.06 | 65 | 59 | 26 | 6 |
| 73 | 2 | 2 | 19.15 | 28.4 | 1.94 | 64 | 90 | 40 | 10 |
| 74 | 2 | 2 | 19.21 | 31.4 | 2.56 | 66 | 49 | 21 | 6 |
| 75 | 2 | 1 | 19.42 | 13.5 | 1.03 | 71 | 37 | 11 | 7 |
| 76 | 2 | 2 | 19.54 | 25.0 | 0.94 | 60 | 67 | 33 | 6 |
| 77 | 2 | 1 | 20.31 | 28.3 | 1.28 | 78 | 39 | 22 | 4 |
| 78 | 2 | 2 | 20.34 | 24.3 | 0.43 | 65 | 33 | 18 | 3 |
| 79 | 2 | 2 | 20.62 | 28.3 | 1.20 | 62 | 22 | 4 | 4 |
| 80 | 2 | 2 | 20.62 | 24.1 | 1.82 | 71 | 64 | 15 | 11 |
| 81 | 2 | 2 | 20.63 | 32.3 | 0.53 | 70 | 50 | 23 | 8 |
| 82 | 2 | 1 | 20.64 | 13.7 | 1.14 | 78 | 68 | 30 | 12 |
| 83 | 2 | 1 | 20.83 | 11.8 | 1.61 | 72 | 31 | 14 | 5 |
| 84 | 2 | 2 | 20.89 | 27.7 | 1.29 | 67 | 58 | 23 | 12 |
| 85 | 2 | 2 | 20.91 | 26.0 | 0.65 | 69 | 64 | 41 | 7 |
| 86 | 2 | 2 | 21.01 | 30.0 | 1.46 | 77 | 52 | 15 | 8 |
| 87 | 2 | 2 | 21.08 | 29.6 | 1.78 | 66 | 66 | 32 | 10 |
| 88 | 2 | 1 | 21.35 | 11.9 | 1.05 | 82 | 54 | 25 | 6 |
| 89 | 2 | 2 | 21.47 | 35.5 | 1.00 | 63 | 92 | 25 | 6 |
| 90 | 2 | 2 | 21.49 | 31.4 | 4.43 | 77 | 41 | 27 | 6 |
| 91 | 2 | 2 | 21.54 | 30.7 | 1.69 | 71 | 42 | 24 | 8 |
| 92 | 2 | 2 | 21.56 | 34.5 | 1.18 | 72 | 68 | 32 | 8 |
| 93 | 2 | 2 | 21.72 | 27.5 | 0.62 | 69 | 36 | 21 | 4 |
| 94 | 2 | 2 | 21.86 | 26.4 | 1.05 | 70 | 71 | 19 | 7 |
| 95 | 2 | 1 | 22.01 | 20.0 | 1.89 | 73 | 35 | 24 | 4 |
| 96 | 2 | 1 | 22.08 | 15.1 | 0.32 | 78 | 48 | 19 | 6 |
| 97 | 2 | 2 | 22.10 | 34.2 | 1.53 | 66 | 67 | 24 | 8 |
| 98 | 2 | 2 | 22.15 | 30.0 | 0.55 | 66 | 96 | 32 | 6 |
| 99 | 2 | 1 | 22.17 | 10.9 | 0.72 | 79 | 53 | 16 | 8 |
| 100 | 2 | 1 | 22.21 | 18.6 | 2.07 | 73 | 65 | 35 | 6 |
| 101 | 2 | 2 | 22.40 | 34.4 | 1.93 | 74 | 60 | 26 | 6 |
| 102 | 2 | 2 | 22.61 | 31.0 | No data | 68 | 55 | 14 | 7 |
| 103 | 2 | 1 | 22.89 | 18.3 | 2.00 | 77 | 83 | 37 | 9 |
| 104 | 2 | 2 | 22.91 | 32.8 | 2.00 | 78 | 48 | 28 | 7 |
| 105 | 2 | 1 | 23.09 | 20.3 | 1.25 | 78 | 64 | 34 | 4 |
| 106 | 2 | 1 | 23.37 | 19.9 | 2.66 | 83 | 73 | 29 | 6 |
| 107 | 2 | 1 | 23.43 | 28.1 | 1.17 | 76 | 70 | 27 | 10 |
| 108 | 2 | 2 | 23.43 | 32.8 | 0.99 | 75 | 72 | 30 | 8 |
| 109 | 2 | 2 | 23.46 | 39.1 | 2.54 | 74 | 57 | 25 | 4 |
| 110 | 2 | 2 | 23.55 | 32.2 | 1.51 | 76 | 49 | 34 | 4 |
| 111 | 2 | 1 | 23.75 | 19.0 | 0.88 | 83 | 45 | 18 | 11 |
| 112 | 2 | 2 | 24.02 | 38.0 | 2.42 | 84 | 43 | 22 | 4 |
| 113 | 2 | 2 | 24.03 | 37.7 | 1.31 | 73 | 58 | 23 | 8 |
| 114 | 2 | 2 | 24.07 | 35.3 | 2.57 | 80 | 32 | 30 | 2 |
| 115 | 2 | 1 | 24.18 | 18.9 | 0.82 | 80 | 56 | 24 | 8 |
| 116 | 2 | 2 | 24.55 | 34.9 | 1.03 | 85 | 64 | 32 | 7 |
| 117 | 2 | 1 | 24.56 | 19.4 | 1.66 | 78 | 40 | 23 | 6 |
| 118 | 2 | 1 | 24.67 | 13.6 | 1.42 | 83 | 53 | 24 | 6 |
| 119 | 2 | 2 | 24.76 | 41.9 | 1.05 | 78 | 43 | 11 | 9 |
| 120 | 2 | 2 | 24.80 | 32.4 | 0.45 | 69 | 72 | 26 | 10 |
| 121 | 2 | 1 | 24.95 | 43.6 | 2.09 | 93 | 16 | 3 | 5 |
| 122 | 2 | 2 | 25.00 | 40.1 | 1.12 | 73 | 34 | 13 | 5 |
| 123 | 2 | 1 | 25.83 | 21.4 | 0.34 | 81 | 47 | 30 | 8 |
| 124 | 2 | 2 | 26.19 | 37.0 | 1.38 | 77 | 53 | 24 | 6 |
| 125 | 2 | 1 | 26.53 | 21.6 | 1.74 | 83 | 53 | 15 | 6 |
| 126 | 2 | 1 | 27.56 | 22.2 | 0.97 | 84 | 40 | 17 | 6 |
| 127 | 2 | 2 | 28.17 | 44.9 | 1.77 | 91 | 72 | 41 | 9 |
| 128 | 2 | 1 | 29.70 | 39.7 | 4.01 | 92 | 46 | 19 | 6 |
| 129 | 2 | 1 | 30.30 | 41.6 | 2.45 | 102 | 40 | 15 | 7 |
| 130 | 2 | 1 | 30.96 | 30.4 | 1.64 | 98 | 48 | 29 | 5 |
| 131 | 2 | 1 | 31.42 | 22.7 | 0.44 | 88 | 52 | 27 | 6 |
| 132 | 2 | 1 | 31.98 | 36.0 | 4.08 | 106 | 61 | 27 | 4 |
